# CD47 Blockade Inhibits Tumor Progression through Promoting Phagocytosis of Tumor Cells by M2 Polarized Macrophages in Endometrial Cancer

**DOI:** 10.1155/2018/6156757

**Published:** 2018-11-07

**Authors:** Shenglan Gu, Ting Ni, Jing Wang, Yao Liu, Qiong Fan, Yiwei Wang, Ting Huang, Yiwei Chu, Xiao Sun, Yudong Wang

**Affiliations:** ^1^Department of Gynecology, International Peace Maternity and Child Health Hospital, Shanghai Jiao Tong University School of Medicine, Shanghai, China; ^2^Department of Immunology, School of Basic Medical Sciences, Fudan University, Shanghai, China; ^3^Biotherapy Research Center, Fudan University, Shanghai, China; ^4^Laboratory of Gynecologic Oncology, International Peace Maternity and Child Health Hospital, Shanghai Jiao Tong University School of Medicine, Shanghai, China

## Abstract

There are rapidly emerging efforts to explore tumor-associated macrophages (TAMs) as a tumor therapy target. Tumor cells express CD47, which can interact with the macrophages' SIRP*α* transmitting a “don't eat me” signal to macrophages. The expression of CD47 increases in various tumors to evade immune attack. However, the expression of CD47 in endometrial cancer (EC) and the role of CD47-SIRP*α* in the TAMs which mediate the progression of EC remain unclear. Our study shows that there are increased TAMs in EC which dominantly consist of M2 macrophages and contribute to the progression of EC. We confirm that CD47 is highly expressed in EC tissue using the TCGA database, qPCR, and flow cytometry. Instead of directly promoting the apoptosis of EC cells, anti-CD47 blocking antibody promoted phagocytosis of EC cells by macrophages and the increased phagocytosis ability was mediated by M2 macrophages in a coculture assay. Besides, CD47 blockade inhibited the growth of the EC tumors *in vivo* and increased the infiltration of macrophages with antitumor ability in the tumor microenvironment (TME). These findings might assist in developing promising strategies that blocked the CD47-SIRPa interaction for EC therapy.

## 1. Introduction

Endometrial cancer is one of the most common gynecological malignancies, with 61,380 estimated new cases and 10,920 estimated deaths in 2017 in America [1]. Patients in less developed regions have poorer prognosis [2]. Novel therapeutic options are desperately needed. Tumor immunotherapies which target the tumor microenvironment to increase the antitumor activity of the immune system elicit durable responses in many kind of tumors [3, 4]. The tumor microenvironment (TME), which is composed of tumor cells, immune cells, tumor-associated fibroblasts, the vascular network, cytokines, and so on [5], tends to be polarized to an immunosuppressive state to facilitate the tumor immune evasion [6]. In endometrial cancer, neoplastic cells can exploit a large variety of immune evasion mechanisms, including alterations in the expression of some molecules that inhibit antitumor immune response, such as programmed cell death 1 ligand 1 (PD-L1) and indoleamine-2,3-dioxygenase (IDO) [7, 8]. Accumulating evidence indicates that anti-PD-1/PD-L immune checkpoint therapy may be effective in DNA polymerase epsilon- (POLE-) mutated and microsatellite instability (MSI) EC patients [9–11]. Considering that POLE-mutated and MSI EC patients account for a small fraction of the total EC population (7%–12% and 20%–30%, respectively) and have better prognosis [12, 13], more universal drugs should be found.

Recently, the role of immune cells in the TME is well demonstrated in tumor progression and immunotherapy [5, 14]. Macrophages infiltrating into the TME are termed the tumor-associated macrophages (TAMs), which are the major component of infiltrating leukocytes in most tumors [15]. Macrophages are characterized by considerable heterogeneity and have been divided into two general subtypes: the classically activated M1 macrophages which have the potential to exhibit antitumor activity, and the alternatively activated M2 macrophages which are considered to be involved in tumor growth and progression [16]. TAMs tend to acquire a polarized M2 phenotype in many kinds of tumors with low antitumor activity through various mechanisms [17]. It is important to investigate the phenotype, phagocytosis ability, and antigen presenting ability of TAMs in EC.

Considering that TAMs contribute to the formation of an immunosuppressed state within the TME, one of the therapeutic strategies targeting TAMs is reeducating TAMs to an antitumor phenotype, such as promoting macrophages' phagocytosis ability [18, 19]. Accumulating evidences show that the CD47-SIRP*α* signal participates in tumor immune evasion mediated by TAMs [20, 21]. CD47 is a broadly expressed membrane protein on various tumor cells and plays an important role in self-recognition by which normal cells protect themselves from phagocytosis [21]. Signal regulatory protein alpha (SIRP*α*, also known as CD172a), which mainly expresses on the surface of macrophages, is the receptor for CD47. When CD47 binds to SIRP*α*, the intracellular immunoreceptor tyrosine-based inhibitory motifs (ITIMs) of SIRPα is phosphorylated, followed by recruitment and activation of the tyrosine phosphatases such as SHP-1 and SHP-2. Then, the phosphoprotein substrates are dephosphorylated which affect downstream signaling pathways, transmitting a “don't eat me” signal to inhibit the macrophages' phagocytosis ability [22]. Accumulating evidences showed that CD47 was upregulated in many malignancies to evade the immune attack, and its overexpression was correlated with poor prognosis [23–26]. Besides, interruption of the ligation of CD47 and SIRP*α* promotes the tumor cells to be phagocytosed by macrophages in various malignancies [24, 27, 28]. A number of different drugs targeting the CD47-SIRPα signal are evaluated in patients with solid tumors in clinical trials (http://clinicaltrials.gov identifiers: NCT02216409, NCT02890368, NCT02953782, and NCT03013218).

Intriguingly, researchers found that CD47 was expressed on all cancer cells from patients [25], pointing out that it is necessary to investigate the expression of CD47 in EC. To our knowledge, the role of the CD47-SIRP*α* signal in EC has not been studied yet. To clarify whether the CD47-SIRP*α* signal contributes to the immune evasion mediated by TAMs, we perform a phagocytosis assay *in vitro* and establish the xenograft EC model to test the antitumor activity of CD47 blockade therapy. Our studies highlight the potential therapeutic strategy in which reeducating TAMs may have beneficial antitumor effects in EC.

## 2. Materials and Methods

### 2.1. Preparation of Tissue Samples

All human samples were obtained from the International Peace Maternity and Child Health Hospital after receiving patients' informed consent.

### 2.2. Immunohistochemistry

The paraffin-embedded tissues were sectioned into 4 *μ*m, then deparaffinized and rehydrated with xylene and graded alcohol. Antigen retrieval was used with EDTA. Sections were incubated with mouse anti-human CD68 antibody (1 : 200; Abcam), mouse anti-human CD163 antibody (1 : 1000; Bio-Rad), and anti-human CD47 antibody (1 : 1000; GeneTex) at 4°C overnight. EXPOSE Mouse and Rabbit Specific HRP/DAB Detection IHC Kit (Abcam) was used for the following steps according to the manufacturer's protocol. All the samples were assessed by two pathologists in 10 different high-power fields (HPFs). The number of CD68^+^ cells and CD163^+^ cells were counted and the average taken. The staining intensity of CD47 was scored as 0 (no staining), 1 (weak staining), 2 (intermediate staining), or 3 (dark staining). The percentage of staining cells was scored as 0 (0–5%), 1 (1–25%), 2 (26–50%), 3 (51–75%), or 4 (76–100%). The product of the two scores were considered as the CD47 IHC score. Samples were classified as low CD47 expression (IHC score ≤ 4) or high CD47 expression (IHC score > 4).

### 2.3. Immunofluorescence

Sections or coculture cell (mouse macrophages + EC cells) dishes were blocked with 10% calf serum and incubated with rabbit anti-human CD68 antibody (1 : 100), mouse anti-human CD163 antibody (1 : 1000), and anti-mouse F4/80 antibody (1 : 200; Abcam) at 4°C overnight. The sections were then incubated with Alexa Fluor 488 donkey anti-mouse IgG, Alexa Fluor 594 donkey anti-mouse IgG, and Alexa Fluor 594 donkey anti-rabbit IgG (Life Technologies) at room temperature for 2 hours followed by nuclear counterstaining with DAPI (Abcam). The samples were detected by confocal microscopy.

### 2.4. RNA Extraction and qPCR

The tissues used for RNA extraction were ground by a TissueLyser. Total RNA was extracted using the TRIzol (Invitrogen) method. cDNA was synthesized from 1 *μ*g total RNA using a reverse transcription kit (Tiangen Biotech (Beijing) Co. Ltd., China). Real-time PCR was performed using the SYBR Green Master Mix (Takara Bio Inc.) on a 7500 Real-Time PCR System (Applied Biosystems). The 2^−ΔΔCt^ method was used to calculate fold changes in the gene expression normalized to GAPDH. The primers that were used are shown Table 1.

### 2.5. The Preparation of Tissue Single-Cell Suspension

From May 2017 to October 2017, 27 patients who underwent hysterectomy for EC or other benign diseases were recruited into this study. Clinical endometrial tissues were obtained from the patients after getting their informed consent. Fresh endometrial tissue specimens were transported on ice to the laboratory, cut into small pieces of 2–4 mm, and enzymatically dissociated with the Tumor Dissociation Kit (Miltenyi Biotec). ACK Lysing Buffer (Thermo Fisher Scientific) was used to remove erythrocytes. Cells were then washed twice with PBS and filtered through a 70 *μ*m filter.

### 2.6. Flow Cytometry Analysis

Single-cell suspensions were stained with FITC-conjugated anti-CD47 (eBioscience). 7-AAD (eBioscience) and antibodies targeted to CD45 (BD Biosciences) and CD31 (eBioscience) were used to exclude dead, nontumor cells. Flow cytometry analyses were performed on BD FACS Canto II.

### 2.7. Murine BMDM Culture and Differentiation

Murine bone marrow cells were collected from 8-week old NOD/SCID/IL2*γ* ^null^ mice (NSG, Beijing Biocytogen Co. Ltd.). 1 × 10^6^ murine bone marrow cells were planted per well in a 24-well plate and cultured with Dulbecco's Modified Eagle Medium (DMEM, Gibco) supplied with recombinant mouse macrophage colony-stimulating factor (M-CSF; 10 ng/mL, R&D Systems) for 7 days. The macrophages at this state were considered as M0 macrophages. Purity was verified by flow cytometry using F4/80 and CD11b. M1 MΦ were obtained by further treatment on day 7 with recombinant mouse interferon-gamma (IFN-*γ*, 20 ng/mL) and lipopolysaccharide (LPS; 100 ng/mL, Sigma-Aldrich) for 24 hours. M2 MΦ were obtained by further treatment on day 7 with IL-4 (20 ng/mL) for 24 hours. All cytokines were purchased from PeproTech Inc. unless otherwise stated.

### 2.8. Establishment of CD47 Knockdown EC Cells

We designed a shRNA to target the human CD47 gene (NM_198793.2). The CD47-shRNA and control sequences are as follows: CD47-shRNA (5′-CCGGGCACAATTACTTGGACTAGTTCTCGAGAACTAGTCCAAGTAATTGTGCTTTTT-3′), scramble-shRNA (5′-CCGGTTCTCCGAACGTGTCACGTTTCAAGAGAACGTGACACGTTCGGAGAA TTTTTG-3′). The shRNA was cloned into a lentiviral vector (pL-TO-IRES-LUC) to knockdown the expression of CD47 in EC cells (GeneChem Biotech, Shanghai, China). 1 × 10^5^ Ishikawa cells or KLE cells were transfected with 2 × 10^6^ TU shRNA-encoding lentivirus in the presence of polybrene (5 *μ*g/mL) for 12 h. Then, the EC cells were cultured in DMEM with 10% FBS for 1 week. Puromycin (1 *μ*g/mL) was used to select the cells that were successfully transfected. After 2 weeks, the CD47 protein expression on EC cells was detected by flow cytometry.

### 2.9. *In Vitro* Phagocytosis Assay

For the *in vitro* phagocytosis assay, Ishikawa cells were labeled with 1 *μ*M CFSE using the CellTrace CFSE Cell Proliferation Kit (Invitrogen). Macrophages were incubated with 1 × 10^6^ CFSE-labeled Ishikawa cells in serum-free medium in the presence of IgG control (10 *μ*g/mL, eBioscience) or anti-CD47 antibodies (10 *μ*g/mL, eBioscience) for 2 h. Then, the plate was washed for 3 times with warm PBS to remove unphagocytosed Ishikawa cells.

For the immunofluorescence assay, the cocultured cells were observed through a fluorescence microscope to investigate the phagocytosis of EC cells by macrophages. For the flow cytometry assay, the cocultured cells were digested with 0.25% Trypsin-EDTA (Gibco). A single-cell suspension was incubated with a mAb specific for mouse CD16/CD32 to prevent nonspecific binding against Fc*γ*R, and it was then incubated with F4/80 antibodies (BioLegend) for 30 min at 4°C and washed twice with 2% FBS in PBS. Stained cells were subjected to flow cytometry, and data was analyzed with FlowJo software. The phagocytic index was calculated as the percentage of CFSE^+^ macrophages.

### 2.10. Apoptosis Assay

The EC cells were treated with anti-CD47 antibody (B6H12; 10 *μ*g/mL) or control antibody (10 *μ*g/mL) for 2 h or 12 h. Then, the EC cells were digested and the apoptosis was measured by flow cytometry using Annexin V-FITC and PI (BD Biosciences).

### 2.11. Tumor Xenograft Assay in NSG Mice

Twenty 7-week old female NSG mice were obtained from the Beijing Biocytogen Co. Ltd. Animal research was carried out in strict accordance with the Guideline for the Care and Use of Laboratory Animals of China. 10^7^ CD47-knockdown Ishikawa cells or control Ishikawa cells were injected subcutaneously to the left flank of NSG mice, and tumor growth was monitored. The tumor volume and body weight were measured per week. After four weeks, the mice were sacrificed by cervical dislocation and tumor bulk was removed from the animals.

### 2.12. Statistical Analysis

Statistical analyses were performed using GraphPad Prism 6.0. Data were analyzed by unpaired Student's *t*-test or one-way ANOVA and were presented as the mean ± SEM. *P* values < 0.05 were considered statistically significant. All experiments were repeated three times.

## 3. Results

### 3.1. M2 TAMs Are Closely Associated with the Tumor Progression in EC

**Figure 1:**
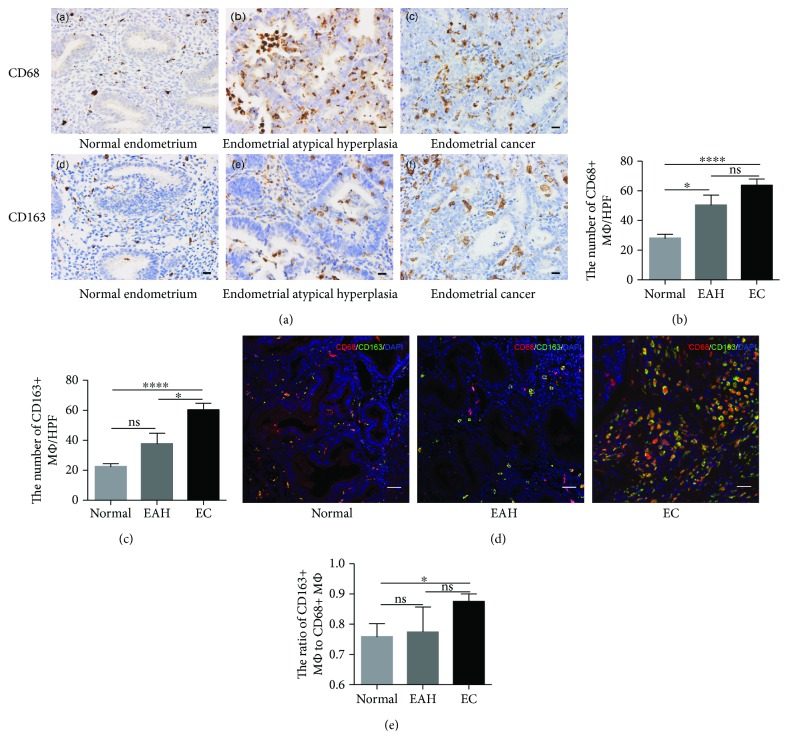
The majority of infiltrated macrophages in EC tissues are M2 TAMs. (a) Representative immunohistochemical staining of CD68 and CD163 (400x) in normal, EAH, and EC tissues. CD68 (a–c) and CD163 (d–f). Scale bars, 20 *μ*m. (b–c) Cell counts of CD68^+^ and CD163^+^ macrophages. (d) Representative images of CD68^+^CD163^−^ macrophages (M1 macrophages) and CD163^+^CD68^+^ macrophages (M2 macrophages). Scale bars, 20 *μ*m. (e) The ratio of CD163^+^ to CD68^+^ cells (M2/total macrophage ratio). There were 26 normal endometrium samples, 11 EAH samples, and 47 EC samples. Data were shown as the mean ± SEM (ns, not significant; ^∗^ *P* < 0.05 and ^∗∗∗∗^ *P* < 0.0001).

To study the distribution of macrophages, immunohistochemistry was used to evaluate the infiltration of TAMs in normal endometrium, endometrial atypical hyperplasia (EAH), and EC (Figure 1(a)). CD68 and CD163 are relatively commonly accepted markers for total macrophages and M2 macrophages, respectively [19]. There were more macrophages infiltrating in EC than in the normal endometrium (Figures 1(b)-1(c)), which were mainly M2 macrophages (Figures 1(d)-1(e)). Besides, there was a progressive upregulation of M2 macrophages from the normal endometrium and EAH to EC (Figure 1(c)).

On the basis of the findings that macrophage infiltration was correlated with patient survival, we hypothesized that macrophage infiltration might play roles in the progression of EC. The relationship between total or M2 macrophages and clinicopathological features was analyzed (Table 2). The high number of macrophages, particularly M2 macrophages in EC, was strongly correlated with unfavorable prognostic factors, such as high pathological grade (*P* = 0.0118), high FIGO stage (*P* < 0.0001), lymph node metastasis (*P* = 0.0008), and lymphovascular space involvement (*P* = 0.0031). Our results showed that M2 TAM infiltration was closely associated with the progression of EC.

### 3.2. CD47 Is Highly Expressed in EC Compared with Normal Endometrium

**Figure 2:**
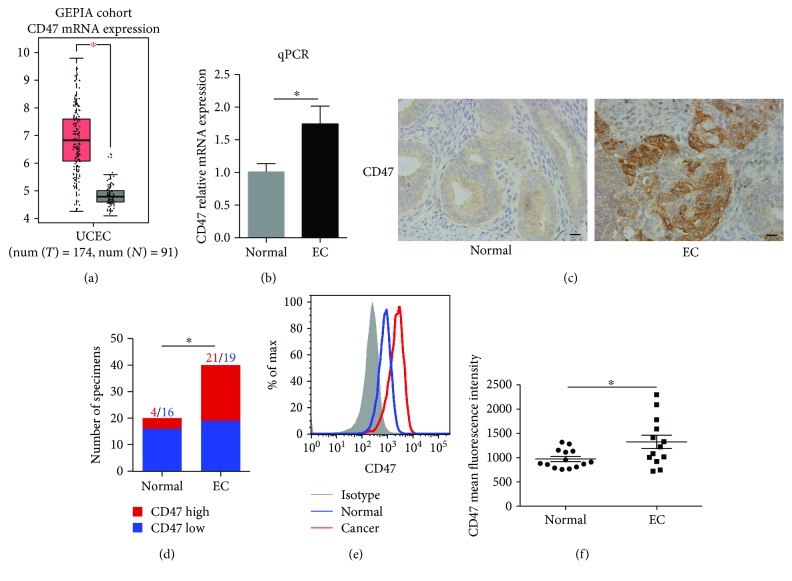
CD47 is highly expressed in EC tissues. (a) Analysis of CD47 mRNA expression in EC samples using TCGA RNAseq. (b) CD47 mRNA expression measured by qPCR. There were 18 normal endometrium samples and 22 EC samples. (c) The representative immunohistochemical staining of CD47 (400x) measured by immunochemistry. Scale bars, 20 *μ*m. (d) The quantification of CD47 expression measured by immunochemistry. There were 20 normal endometrium samples and 40 EC samples. (e) The representative image of CD47 expression measured by flow cytometry. (f) The quantification of CD47 expression measured by flow cytometry. There were 14 normal endometrium samples and 13 EC samples. Data were shown as the mean ± SEM (^∗^ *P* < 0.05).

Previous researches have reported that CD47 was overexpressed in various tumors [23–28]. We found that CD47 mRNA was highly expressed in EC samples using The Cancer Genome Atlas Research Network (TCGA) database (Figure 2(a)). CD47 was highly expressed in EC tissue when analyzed by qPCR (Figure 2(b)). CD47 protein expression level was increased in EC tissues by immunochemistry (Figures 2(c)-2(d)). Considering CD47 that expressed on the cell surface interacted with SIRP*α*, we evaluated the CD47 expression on freshly isolated cells from EC tissue and normal endometrium by flow cytometry. Although CD47 protein was detectable on all specimens, it was significantly overexpressed in tumor tissue compared with normal tissue (Figure 2(e)). Besides, CD47 was detectable in all EC cell lines that we tested (Supplementary Figure 1).

### 3.3. CD47 Blockade Increases Phagocytosis of EC Cells by Macrophages *In Vitro*

**Figure 3:**
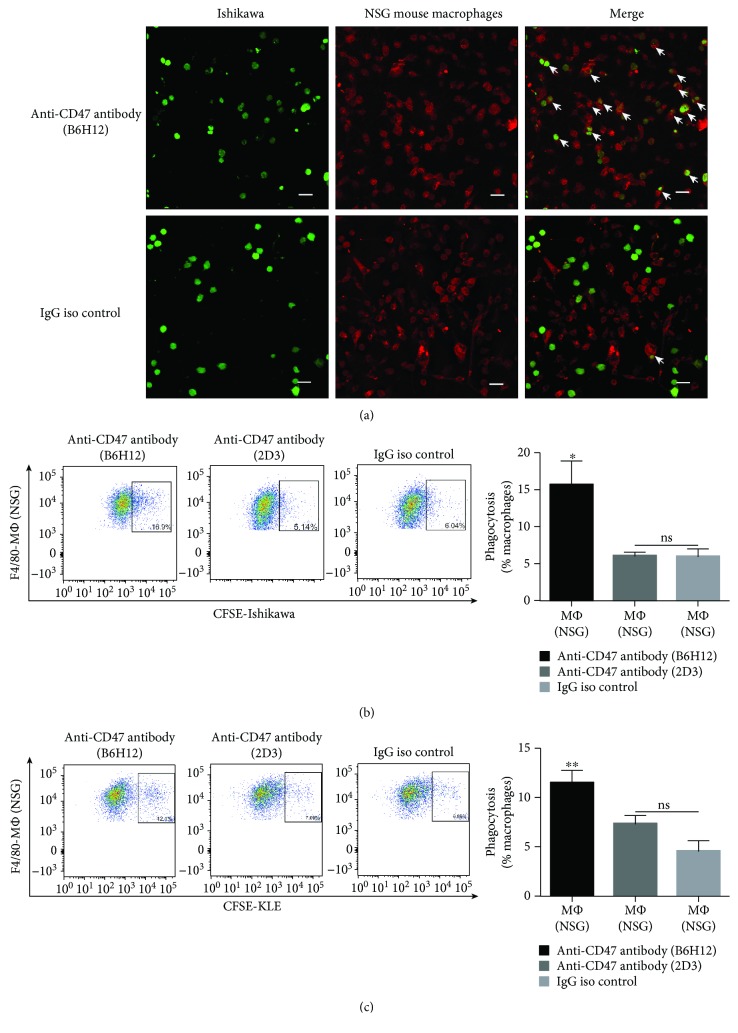
CD47 blockade increases phagocytosis of EC cells by macrophages. (a) Representative images of the phagocytosis assay in which Ishikawa cells were cocultured with NSG mouse BMDMs in the presence of anti-CD47 antibody or control IgG antibody. The white arrows point to the macrophages that phagocytosed Ishikawa cells. Scale bars, 20 *μ*m. (b-c) Flow cytometry results of phagocytosis assays in which Ishikawa or KLE cells were cocultured with NSG mouse BMDMs. Percentages of CFSE^+^ F4/80^+^ macrophages in total macrophages were indicated beside the gated population. Data were shown as the mean ± SEM (ns, not significant; ^∗^ *P* < 0.05 and ^∗∗^ *P* < 0.01).

To directly study the inhibitory effect of the interaction between CD47 and SIRP*α*, we performed phagocytosis assays *in vitro*. The majority of the NSG or C57BL/6 mouse bone marrow-derived macrophages (BMDMs) were CD11b^+^F4/80^+^ macrophages which suggested successful cultivation (Supplementary Figure 2A). NSG mouse BMDMs were cocultured with EC cells (Ishikawa cells or KLE cells) with or without the anti-CD47 antibody. Phagocytosis was evaluated by the percentage of macrophages engulfing EC cells. CD47 blockade with the anti-CD47 blocking antibody (B6H12) resulted in a significant increase in phagocytosis of Ishikawa cells by NSG and C57BL/6 mouse macrophages, while this effect was not observed with the anti-CD47 nonblocking antibody (2D3), which was specific to CD47 but did not interrupt the interaction between CD47 and SIRP*α* (Figures 3(a)-3(b), Supplementary Figures 2B-2C). CD47 blockade could increase phagocytosis of KLE cells by NSG BMDMs (Figure 3(c)). Some studies showed that anti-CD47 antibodies might directly induce the apoptosis of tumor cells [29, 30]. However, our results showed that the soluble anti-CD47 antibody (B6H12) could not promote the apoptosis of EC cells (Supplementary Figure 3).

### 3.4. CD47 Knockdown Increases Phagocytosis of EC Cells by Macrophages *In Vitro*

**Figure 4:**
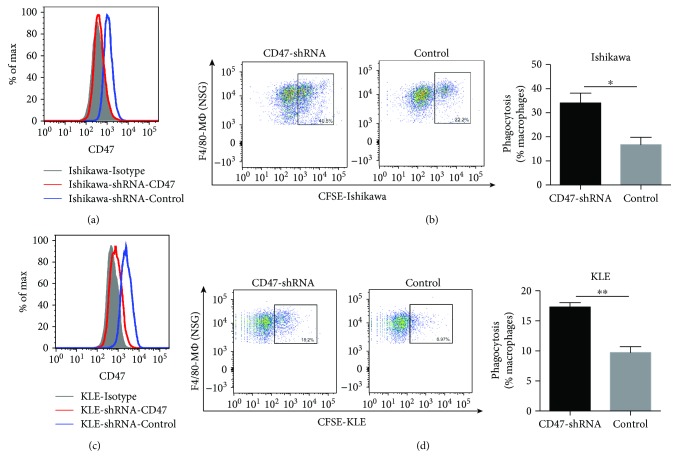
CD47 knockdown increases phagocytosis of EC cells by macrophages. (a, c) Successful knockdown of CD47 in EC cell lines (Ishikawa cell line and KLE cell line) measured by flow cytometry. (b, d) Representative images of the phagocytosis assay in which CD47-knockdown EC cells (Ishikawa or KLE cells) or control EC cells were cocultured with NSG mouse BMDMs. Percentages of CFSE^+^ F4/80^+^ macrophages in total macrophages were indicated beside the gated population. Data are shown as the mean ± SEM (ns, not significant; ^∗^ *P* < 0.05 and ^∗∗^ *P* < 0.01).

We performed a CD47-knockdown experiment in Ishikawa cells and KLE cells using a lentiviral-based approach and confirmed that the successful establishment of CD47-knockdown EC cells (Figures 4(a), 4(c)). There was an increase in phagocytosis to both CD47-knockdown Ishikawa cells and CD47-knockdown KLE cells by NSG mouse BMDMs in phagocytosis assays (Figures 4(b), 4(d)).

### 3.5. The Increased Phagocytosis Ability with CD47 Blockade Treatment Is Mediated by M2 Macrophages *In Vitro*

**Figure 5:**
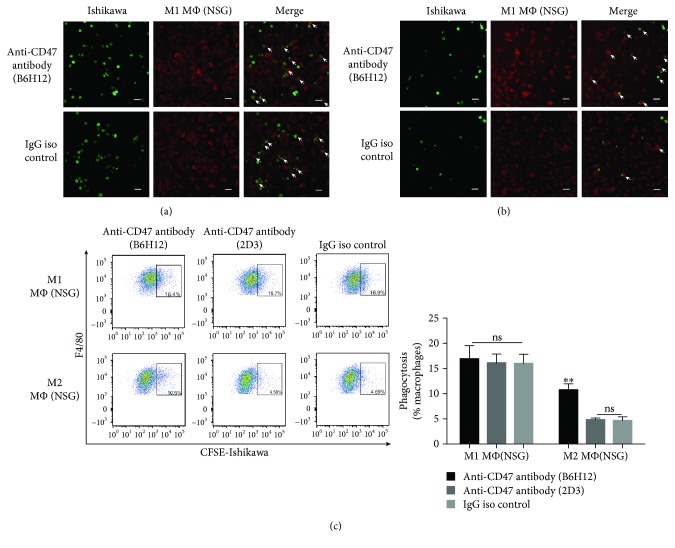
CD47 blockade increases phagocytosis of EC cells by M2 macrophages. (a-b) Representative images of the phagocytosis assay in which Ishikawa cells were cocultured with M1 or M2 NSG mouse BMDMs in the presence of anti-CD47 antibody or control IgG antibody. The white arrows point to the macrophages that phagocytosed Ishikawa cells. Scale bars, 20 *μ*m. (c) Flow cytometry results of phagocytosis assays. Percentages of CFSE^+^ F4/80^+^ macrophages in total macrophages were indicated beside the gated population. Data were shown as the mean ± SEM (ns, not significant; ^∗∗^ *P* < 0.01).

To study whether the CD47 blockade can influence the phagocytosis of the macrophages with different states, we induced NSG mouse BMDMs into different phenotypes (Supplementary Figure 4). Then, the polarized macrophages were cocultured with Ishikawa cells in the presence of anti-CD47 blocking, nonblocking antibodies, and control IgG antibody. In the control group, we found that M1 macrophages had a greater ability of phagocytosis, compared to M2 macrophages (Figures 5(a)-5(c)). However, we found that M2 macrophages display a larger phagocytic response towards EC cells than M1 macrophages when treated with anti-CD47 blocking antibody (Figures 5(a)-5(c)). In other words, CD47^+^ Ishikawa cells mainly inhibited themselves from being engulfed by M2 macrophages rather than by M1 macrophages.

### 3.6. CD47 Knockdown Inhibits Tumor Growth and Promotes the Infiltration of M1 Macrophages in the TME *In Vivo*

**Figure 6:**
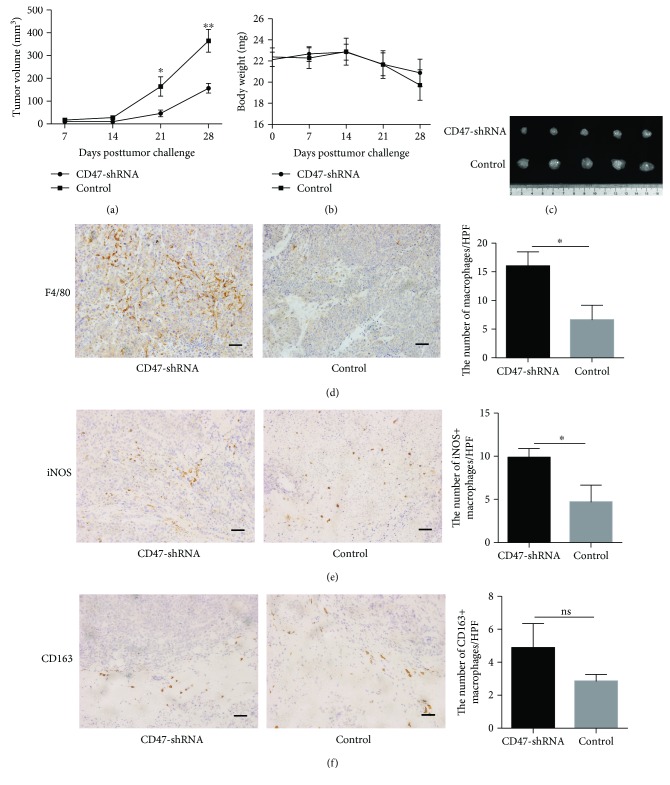
CD47 knockdown inhibits EC growth *in vivo* and promotes the infiltration of M1 macrophages in tumors. (a) Tumor volumes of heterotopic xenografted tumors in NSG mice which were engrafted with CD47-knockdown Ishikawa cells or control Ishikawa cells measured every 7 days. (b) The body weight of the mice measured every 7 days. (c) Tumor volumes of different groups. (d) Representative images and cell counts of F4/80^+^ macrophages in tumor mass (200x). Scale bars, 50 *μ*m. (e) Representative images and cell counts of iNOS^+^ macrophages in tumor mass (200x). Scale bars, 50 *μ*m. (f) Representative images and cell counts of CD163^+^macrophages in tumor mass (200x). Scale bars, 50 *μ*m. Data are shown as the mean ± SEM (ns, not significant; ^∗^ *P* < 0.05 and ^∗∗^ *P* < 0.01).

To examine whether CD47 knockdown contributed to the growth of EC, we found that the size of the tumors formed by shCD47 clones in NSG mice were smaller, when compared with those in the control group (Figures 6(a), 6(c)), although there was no difference in mouse body weight between the two groups (Figure 6(b)). More importantly, we observed that there were more macrophages which were mainly M1 macrophages in xenografted tumors formed by shCD47 clones than in the control group (Figures 6(d)-6(f)). These results suggested that CD47 knockdown inhibited the growth of the EC tumors *in vivo* and promoted the infiltration of macrophages which might play an important role in antitumor activity.

## 4. Discussion

The underlying mechanism of tumor progression and immune evasion mediated by TAMs in EC has been poorly characterized to date. Our results showed that TAMs in EC tended to acquire a polarized M2 phenotype which might contribute to skewing the TME to a tumor-progressive condition. Besides, our results indicated that the increased number of TAMs was positively correlated with the progression of EC; therefore, they are consistent with other studies [31–33].

CD47 expressed by tumor cells interact with SIRP*α* transmitting a “don't eat me” signal to macrophages to avoid being eliminated. The CD47 overexpression was responsible for immune suppression and tumor progression in EC. The CD47 blockade treatment could increase the phagocytosis ability of M2 macrophages instead of M1 macrophages *in vitro*. These results suggested that CD47 blockade therapy could take advantage of M2 macrophages in the TME without affecting the normal function of M1 macrophages in our bodies. Besides, the *in vivo* experiment suggested that there were smaller tumor sizes and increased TAMs which dominantly consisted of M1 macrophages in the CD47-knockdown group, indicating that these macrophages played an important role in eliminating EC cells.

Our study first revealed that CD47 was overexpressed in several EC cell lines and in all clinical specimens that we tested, regardless of pathological or molecular features. Besides, the anti-CD47 antibody could increase phagocytosis of both Ishikawa cells and KLE cells. Compared to PD-1 blockade immunotherapy which might be effective in a minority of EC patients with the polymerase epsilon (POLE) or microsatellite instability (MSI) mutations [34–36], CD47 blockade immunotherapy might be an extensive and effective choice for EC patients.

In addition to inhibiting the “don't eat me” signal, other potential mechanisms also contributed to the antitumor effects of the anti-CD47 therapy. For instance, some anti-CD47 antibodies could induce the apoptosis of tumor cells directly in several malignancies. However, our results suggested that the antitumor activity of the anti-CD47 blocking antibody was mediated by the interruption of CD47-SIRP*α* interaction instead of promoting the apoptosis of EC cells.

With the use of immunodeficient NSG mice completely lacking T cells, B cells, and NK cells [37], the involvement of macrophages might be the predominant mechanism to regulate the growth of EC *in vivo*. Besides, the SIRP*α* protein produced by NSG mice has greater reactivity with human CD47 than other strains [38] which could better reflect the effect of the CD47-SIRP*α* interaction. However, CD47 negatively regulates the function of the human T cell, dendritic cell [39, 40], NK cell [41], and B cell [42] and plays an inhibitory role in the immune response against tumor cells. The animal model we used could not reflect the role of CD47 in these immune cells. Thus, further studies which focus on CD47 in other immune cells in EC are needed.

## 5. Conclusions

Taken together, we have found that the overexpression of CD47 in EC protected tumor cells against phagocytosis by macrophages *in vitro* and promoted the progression of EC *in vivo*. In conclusion, the CD47 blockade therapy, which can reeducate M2 macrophages by increasing their phagocytosis ability, might be an attractive target for tumor immunotherapy for EC.

## Figures and Tables

**Table 1:** The primers used in this study.

Primers		
CD47 (human)	Sense primer	5′-AGAAGGTGAAACGATCATCGAGC-3′
	Antisense primer	5′-CTCATCCATACCACCGGATCT-3′
GAPDH (human)	Sense primer	5′-ACCACAGTCCATGCCATCAC-3′
	Antisense primer	5′-TCCACCACCCTGTTGCTGTA-3′
TNF-*α* (mouse)	Sense primer	5′-GATCTCAAAGACAACCAACTAGTG-3′
	Antisense primer	5′-AGGTCCAGACGCAGGATGGCATG-3′
iNOS (mouse)	Sense primer	5′-GGCAGCCTGTGAGACCTTTG-3′
	Antisense primer	5′-TGAAGCGTTTCGGGATCTG-3′
IL-12 (mouse)	Sense primer	5′-AAATGAAGCTCTGCATCCTGC-3′
	Antisense primer	5′-TCACCCTGTTGATGGTCACG-3′
Ym1 (mouse)	Sense primer	5′-TCTGGTGAAGGAAATGCGTAAA-3′
	Antisense primer	5′-GCAGCCTTGGAATGTCTTTCTC-3′
Fizz1 (mouse)	Sense primer	5′-CAGCTGATGGTCCCAGTGAA-3′
	Antisense primer	5′-TTCCTTGACCTTATTCTCCACGAT-3′
GAPDH (mouse)	Sense primer	5′-AGGTCGGTGTGAACGGATTTG-3′
	Antisense primer	5′-TGTAGACCATGTAGTTGAGGTCA-3′

**Table 2:** Correlation between the number of CD68^+^ or CD163^+^ macrophages and relevant clinical characteristics of the EC cases.

Parameters	Patients (*n*)	Patients (%)	CD68^+^ macrophages		CD163^+^ macrophages	
			Mean ± SEM	*P* value	Mean ± SEM	*P* value
*Total age (year)*	47	100				
<55	16	34.0	63.37 ± 6.261	0.9676	62.44 ± 6.602	0.7301
≥55	31	66.0	63.73 ± 5.684		58.95 ± 6.336	
*Grade (endometrioid = 37)*	37					
G1 or G2	33	88.6	63.45 ± 5.344	0.1247	56.40 ± 4.675	0.0118^a^
G3	4	11.4	88.98 ± 15.07		99.65 ± 29.49	
*FIGO stage*						
I or II	26	55.3	51.19 ± 5.009	0.0007^a^	44.71 ± 3.859	< 0.0001^a^
III or IV	21	44.7	78.83 ± 5.731		79.24 ± 7.620	
*Histologic type*						
Endometrioid	37	78.7	65.04 ± 4.977	0.507	60.65 ± 5.361	0.8364
Nonendometrioid	10	21.3	57.98 ± 7.415		58.24 ± 10.32	
*Myometrial invasion*						
<1/2	31	66	62.04 ± 5.638	0.6016	58.05 ± 5.843	0.5433
≥1/2	16	34	66.81 ± 5.857		64.18 ± 8.107	
*Positive lymph nodes*						
No	33	64.1	54.75 ± 5.356	0.0042^a^	47.87 ± 4.430	0.0008^a^
Yes	14	35.9	81.63 ± 6.996		84.15 ± 10.81	
*Lymphovascular space involvement*						
No	25	53.2	52.27 ± 5.558	0.0033^a^	47.48 ± 4.536	0.0031^a^
Yes	22	46.8	76.40 ± 5.419		74.53 ± 7.646	

^a^ *P* < 0.05, the difference between CD68/CD163 expression in patients and different grades, FIGO stages, with or without lymph node metastasis, or lymphovascular space involvement.

## Data Availability

The TCGA database analyzed during the current study is available in the Gepia website (http://gepia.cancer-pku.cn). Other data used to support the findings of this study can be accessed by requesting the corresponding author through email (sunxiao000304@163.com).

## Supplementary Materials

Supplementary Materials Supplementary Figure 1: the EC cell lines stably expressed CD47. Supplementary Figure 2: the maturation of mouse BMDMs and CD47 blockade increases phagocytosis of EC cells by C57BL/6 mouse BMDMs. Supplementary Figure 3: anti-CD47 antibody cannot promote apoptosis of EC cells. Supplementary Figure 4: NSG mouse BMDMs are polarized to different phenotypes.

## References

[1] Siegel R. L., Miller K. D., Jemal A. (2017). Cancer. Statistics, 2017. *CA: a Cancer Journal for Clinicians*.

[2] Ferlay J., Soerjomataram I., Dikshit R. (2015). Cancer incidence and mortality worldwide: sources, methods and major patterns in GLOBOCAN 2012. *International Journal of Cancer*.

[3] Gotwals P., Cameron S., Cipolletta D. (2017). Prospects for combining targeted and conventional cancer therapy with immunotherapy. *Nature Reviews. Cancer*.

[4] Tang H., Qiao J., Fu Y. X. (2016). Immunotherapy and tumor microenvironment. *Cancer Letters*.

[5] Quail D. F., Joyce J. A. (2013). Microenvironmental regulation of tumor progression and metastasis. *Nature Medicine*.

[6] Gajewski T. F., Meng Y., Blank C. (2006). Immune resistance orchestrated by the tumor microenvironment. *Immunological Reviews*.

[7] Vanderstraeten A., Luyten C., Verbist G., Tuyaerts S., Amant F. (2014). Mapping the immunosuppressive environment in uterine tumors: implications for immunotherapy. *Cancer Immunology, Immunotherapy*.

[8] Mills A., Zadeh S., Sloan E., Chinn Z., Modesitt S. C., Ring K. L. (2018). Indoleamine 2, 3-dioxygenase in endometrial cancer: a targetable mechanism of immune resistance in mismatch repair-deficient and intact endometrial carcinomas. *Modern Pathology*.

[9] Gargiulo P., Della Pepa C., Berardi S. (2016). Tumor genotype and immune microenvironment in POLE-ultramutated and MSI-hypermutated endometrial cancers: new candidates for checkpoint blockade immunotherapy?. *Cancer Treatment Reviews*.

[10] Nebot-Bral L., Brandao D., Verlingue L. (2017). Hypermutated tumours in the era of immunotherapy: the paradigm of personalised medicine. *European Journal of Cancer*.

[11] Piulats J. M., Matias-Guiu X. (2016). Immunotherapy in endometrial cancer: in the nick of time. *Clinical Cancer Research*.

[12] van Gool I. C., Eggink F. A., Freeman-Mills L. (2015). *POLE* proofreading mutations elicit an antitumor immune response in endometrial cancer. *Clinical Cancer Research*.

[13] Karamurzin Y., Rutgers J. K. L. (2009). DNA mismatch repair deficiency in endometrial carcinoma. *International Journal of Gynecological Pathology*.

[14] Kitamura T., Qian B. Z., Pollard J. W. (2015). Immune cell promotion of metastasis. *Nature Reviews Immunology*.

[15] Belgiovine C., D’Incalci M., Allavena P., Frapolli R. (2016). Tumor-associated macrophages and anti-tumor therapies: complex links. *Cellular and Molecular Life Sciences*.

[16] Sica A., Mantovani A. (2012). Macrophage plasticity and polarization: in vivo veritas. *The Journal of Clinical Investigation*.

[17] Mantovani A., Sozzani S., Locati M., Allavena P., Sica A. (2002). Macrophage polarization: tumor-associated macrophages as a paradigm for polarized M2 mononuclear phagocytes. *Trends in Immunology*.

[18] Bronte V., Murray P. J. (2015). Understanding local macrophage phenotypes in disease: modulating macrophage function to treat cancer. *Nature Medicine*.

[19] Mantovani A., Marchesi F., Malesci A., Laghi L., Allavena P. (2017). Tumour-associated macrophages as treatment targets in oncology. *Nature Reviews. Clinical Oncology*.

[20] Takizawa H., Manz M. G. (2007). Macrophage tolerance: CD47-SIRP-α-mediated signals matter. *Nature Immunology*.

[21] Liu X., Kwon H., Li Z., Fu Y. X. (2017). Is CD47 an innate immune checkpoint for tumor evasion?. *Journal of Hematology & Oncology*.

[22] Barclay A. N., Van den Berg T. K. (2014). The interaction between signal regulatory protein alpha (SIRPα) and CD47: structure, function, and therapeutic target. *Annual Review of Immunology*.

[23] Majeti R., Chao M. P., Alizadeh A. A. (2009). CD47 is an adverse prognostic factor and therapeutic antibody target on human acute myeloid leukemia stem cells. *Cell*.

[24] Chao M. P., Alizadeh A. A., Tang C. (2011). Therapeutic antibody targeting of CD47 eliminates human acute lymphoblastic leukemia. *Cancer Research*.

[25] Willingham S. B., Volkmer J. P., Gentles A. J. (2012). The CD47-signal regulatory protein alpha (SIRPa) interaction is a therapeutic target for human solid tumors. *Proceedings of the National Academy of Sciences of the United States of America*.

[26] Yoshida K., Tsujimoto H., Matsumura K. (2015). CD47 is an adverse prognostic factor and a therapeutic target in gastric cancer. *Cancer Medicine*.

[27] Chao M. P., Alizadeh A. A., Tang C. (2010). Anti-CD47 antibody synergizes with rituximab to promote phagocytosis and eradicate non-Hodgkin lymphoma. *Cell*.

[28] Kim D., Wang J., Willingham S. B., Martin R., Wernig G., Weissman I. L. (2012). Anti-CD47 antibodies promote phagocytosis and inhibit the growth of human myeloma cells. *Leukemia*.

[29] Kikuchi Y., Uno S., Kinoshita Y. (2005). Apoptosis inducing bivalent single-chain antibody fragments against CD47 showed antitumor potency for multiple myeloma. *Leukemia Research*.

[30] Cioffi M., Trabulo S., Hidalgo M. (2015). Inhibition of CD47 effectively targets pancreatic cancer stem cells via dual mechanisms. *Clinical Cancer Research*.

[31] Kübler K., Ayub T. H., Weber S. K. (2014). Prognostic significance of tumor-associated macrophages in endometrial adenocarcinoma. *Gynecologic Oncology*.

[32] Ning C., Xie B., Zhang L. (2016). Infiltrating macrophages induce ERα expression through an IL17A-mediated epigenetic mechanism to sensitize endometrial cancer cells to estrogen. *Cancer Research*.

[33] Soeda S., Nakamura N., Ozeki T. (2008). Tumor-associated macrophages correlate with vascular space invasion and myometrial invasion in endometrial carcinoma. *Gynecologic Oncology*.

[34] Mehnert J. M., Panda A., Zhong H. (2016). Immune activation and response to pembrolizumab in POLE-mutant endometrial cancer. *The Journal of Clinical Investigation*.

[35] Gadducci A., Guerrieri M. E. (2017). Immune checkpoint inhibitors in gynecological cancers: update of literature and perspectives of clinical research. *Anticancer Research*.

[36] Santin A. D., Bellone S., Buza N. (2016). Regression of chemotherapy-resistant polymerase ε (POLE) ultra-mutated and MSH6 hyper-mutated endometrial tumors with Nivolumab. *Clinical Cancer Research*.

[37] Shultz L. D., Lyons B. L., Burzenski L. M. (2005). Human lymphoid and myeloid cell development in NOD/LtSz-scid IL2R gamma null mice engrafted with mobilized human hemopoietic stem cells. *Journal of Immunology*.

[38] Yamauchi T., Takenaka K., Urata S. (2013). Polymorphic Sirpa is the genetic determinant for NOD-based mouse lines to achieve efficient human cell engraftment. *Blood*.

[39] Latour S., Tanaka H., Demeure C. (2001). Bidirectional negative regulation of human T and dendritic cells by CD47 and its cognate receptor signal-regulator protein-alpha: down-regulation of IL-12 responsiveness and inhibition of dendritic cell activation. *Journal of Immunology*.

[40] Tseng D., Volkmer J. P., Willingham S. B. (2013). Anti-CD47 antibody-mediated phagocytosis of cancer by macrophages primes an effective antitumor T-cell response. *Proceedings of the National Academy of Sciences of the United States of America*.

[41] Kim M. J., Lee J. C., Lee J. J. (2008). Association of CD47 with natural killer cell-mediated cytotoxicity of head-and-neck squamous cell carcinoma lines. *Tumour Biology*.

[42] Yoshida H., Tomiyama Y., Ishikawa J. (2000). Integrin-associated protein/CD47 regulates motile activity in human B-cell lines through CDC42. *Blood*.

